# Thermal Manipulation during Embryogenesis Has Long-Term Effects on Muscle and Liver Metabolism in Fast-Growing Chickens

**DOI:** 10.1371/journal.pone.0105339

**Published:** 2014-09-02

**Authors:** Thomas Loyau, Sonia Métayer-Coustard, Cécile Berri, Sabine Crochet, Estelle Cailleau-Audouin, Mélanie Sannier, Pascal Chartrin, Christophe Praud, Christelle Hennequet-Antier, Nicole Rideau, Nathalie Couroussé, Sandrine Mignon-Grasteau, Nadia Everaert, Michel Jacques Duclos, Shlomo Yahav, Sophie Tesseraud, Anne Collin

**Affiliations:** 1 INRA, UR83 Recherches Avicoles, Nouzilly, France; 2 KU Leuven, Department of Biosystems, Leuven, Belgium; 3 University of Liège, Gembloux Agro-Bio Tech, Animal Science Unit, Gembloux, Belgium; 4 Institute of Animal Science, The Volcani Center, Bet Dagan, Israel; University of New England, Australia

## Abstract

Fast-growing chickens have a limited ability to tolerate high temperatures. Thermal manipulation during embryogenesis (TM) has previously been shown to lower chicken body temperature (T_b_) at hatching and to improve thermotolerance until market age, possibly resulting from changes in metabolic regulation. The aim of this study was to evaluate the long-term effects of TM (12 h/d, 39.5°C, 65% RH from d 7 to 16 of embryogenesis vs. 37.8°C, 56% RH continuously) and of a subsequent heat challenge (32°C for 5 h at 34 d) on the mRNA expression of metabolic genes and cell signaling in the *Pectoralis major* muscle and the liver. Gene expression was analyzed by RT-qPCR in 8 chickens per treatment, characterized by low T_b_ in the TM groups and high T_b_ in the control groups. Data were analyzed using the general linear model of SAS considering TM and heat challenge within TM as main effects. TM had significant long-term effects on thyroid hormone metabolism by decreasing the muscle mRNA expression of deiodinase DIO3. Under standard rearing conditions, the expression of several genes involved in the regulation of energy metabolism, such as transcription factor PGC-1α, was affected by TM in the muscle, whereas for other genes regulating mitochondrial function and muscle growth, TM seemed to mitigate the decrease induced by the heat challenge. TM increased DIO2 mRNA expression in the liver (only at 21°C) and reduced the citrate synthase activity involved in the Krebs cycle. The phosphorylation level of p38 Mitogen-activated-protein kinase regulating the cell stress response was higher in the muscle of TM groups compared to controls. In conclusion, markers of energy utilization and growth were either changed by TM in the *Pectoralis major* muscle and the liver by thermal manipulation during incubation as a possible long-term adaptation limiting energy metabolism, or mitigated during heat challenge.

## Introduction

Increased ambient temperature is one of the major constraints for poultry production causing lower productivity, morbidity and mortality and thus leading to economic loss [1]. European and American fast-growing strains of chickens are the main genotypes used in meat-type poultry production worldwide and they exhibit limited ability to tolerate high environmental temperatures, probably because of poorer development of cardio-vascular and respiratory organs compared to muscle [2].

Exposure of fast-growing chickens to heat induces several physiological and behavioral readjustments aimed at restoring homeostasis by reducing their resting metabolic rate [3]. Acute heat exposure induces hyperthermia [4] and causes changes in respiratory physiology and plasma ion concentrations [5], affects the thyroid axis, and increases stress markers [6] and oxidative stress in mitochondria [7]. These changes can cause metabolic disorders and may lead to a cascade of irreversible thermoregulatory events and finally death.

Different strategies have been established in order to reduce the potentially negative impact of heat exposure, including thermal manipulation during embryogenesis (TM). The technique of Piestun et al. [6] consisted of increasing the incubation temperature and relative humidity (RH) from 37.8°C and 56% RH to 39.5°C and 65% RH, 12 h/d from embryonic day (E)7 to E16. This treatment had no effect on hatching parameters but had long-term consequences for chicken physiology. It improved their acquisition of thermotolerance by reducing body temperature in the long term and the mortality of male chickens during heat challenge [6], [8]–[9]. A lower T_b_ has been associated with a better ability to adapt to heat exposure [10].

At d 34 post-hatching, the glycaemia level of TM broilers submitted to a heat challenge was increased, and TM had significant effects on respiratory parameters, increasing O_2_ saturation percentage and decreasing CO_2_ partial pressure in venous blood [9]. The mechanisms underlying this acquisition of thermotolerance have hardly been explored. It has been shown that heat manipulation during embryogenesis reduces O_2_ consumption and heart rate in embryos, suggesting a lower resting metabolic rate and changes in the vasomotor response [11]–[12]. It has also been shown to enhance breast meat yield and decrease abdominal fat pad content [9], [13]. However, the molecular mechanisms underlying such changes in metabolic rate and body composition of TM animals have not been identified to date. Muscle, a major tissue for metabolic heat production in endotherms [14]–[15], and liver, a major site of lipogenesis in avian species [16], warrant specific attention. We therefore investigated the effects of TM during embryogenesis on the expression of genes and activity of enzymes regulating energy (cell signaling, mitochondrial functions…) and protein (proteolysis and protein synthesis…) metabolisms in the *Pectoralis major* (PM) muscle and livers of chickens reared in standard conditions or exposed to a heat challenge at marketing age. Tissue samples were obtained from the TM broilers with lowest body temperature and control broilers with highest body temperature.

## Materials and Methods

### Chemicals

Nitrocellulose membrane, polyacrylamide solution and protein standards were purchased from Bio-Rad Laboratories (Hercules, CA, USA). Antibodies against phospho-extracellular signal-regulated protein kinase p-ERK [T202/Y204], phospho-AMP-activated protein kinase p-AMPK [T172], phospho-p38 mitogen-activated protein (MAP) kinase p-p38 [T180/Y182], phospho-ribosomal protein S6 p-S6 [S235/S236] and S6 were obtained from Cell Signaling Technology (Beverly, MA, USA). Anti-p70S6 kinase or 70 kDa ribosomal protein S6 kinase S6K1 [T389], anti-p38α and anti-ERKα antibodies were from Santa Cruz Biotechnology (Santa Cruz, CA, USA), anti-vinculin from Sigma Chemical Company (St Louis, MO, USA) and anti-AMPKα from Millipore (Paris, France). Alexa Fluor secondary antibodies were purchased from Molecular Probes (Invitrogen, Carlsbad, CA, USA).

### Experimental design

All experiments were carried out in accordance with the legislation governing the ethical treatment of animals and approved by the Ethics Committee (“Comité d'Ethique en Expérimentation Animale Val de Loire”, Tours, France, N° 2011-9).

One thousand Cobb 500 broiler breeder eggs were incubated in semi-commercial incubators (type 360 E, SMA Coudelou, Rochecorbon, France). Control eggs (C) were maintained at 37.8°C and 56% relative humidity (RH) during the whole incubation period [17]. Thermal manipulation treatment (TM) was applied at 39.5°C and 65% RH for 12 h/d from embryonic day (E)7 to E16 [6]. All eggs were turned through 90° every hour. At hatching, chicks were distributed in floor pens at 33°C and the temperature was gradually decreased to 21°C at d 25 and were maintained at 21°C until d 34. Water and standard feeds were supplied *ad libitum*.

At d 32, 375 C and 363 TM animals were divided into heat-challenged and non-challenged sub-treatments. Chickens of the heat-challenged group (CCh and TMCh, respectively) were exposed at d 34 to 32°C for 5 hours, whereas non-challenged chickens remained under standard conditions (C and TM groups, respectively). The average T_b_ of the groups was 40.9±0.1°C (n = 10), 40.7±0.1°C (n = 11), 42.6±0.2°C (n = 10) and 42.6±0.2°C (n = 9), for C, TM, CCh and TMCh, respectively [9]. In earlier studies, Piestun et al. [6], [8] reported lower T_b_ in TM animals than in controls reared in standard conditions and chicken with the lowest T_b_ were shown to have a better ability to adapt to high ambient temperature [10]. The regulation of energy and protein metabolism was therefore investigated in subsets of 8 birds among 9 to 11 per group per treatment exhibiting the lowest body temperatures in the TM groups (potentially the best acclimated) and the highest temperatures in the control groups to highlight thermoregulatory differences (C: 41.1±0.1°C, TM: 40.6±0.2°C (incubation effect: *P*<0.05), CCh: 42.8±0.1°C; TMCh: 42.5±0.2°C (incubation effect: non-significant) [9]. Chickens were slaughtered by cervical dislocation at d 34.

### Tissue sampling

To characterize the pattern of expression of different candidate genes, glycolytic breast PM muscle and livers were removed and snap frozen at d 34 of age in 8 males per treatment. Breast muscle (representing 21% of body mass [9]) was studied in view of the importance of muscle mass in generating metabolic heat. The liver was chosen as a major organ regulating metabolism in birds and mammals, with an additional role in lipogenesis.

### RNA extraction, reverse transcription and qPCR

RNA was simultaneously treated with DNAse and Proteinase K, and extracted from both types of tissue using the Qiagen RNAeasy mini kit (Qiagen, The Netherlands) according to the manufacturer's instructions. The amounts and purity of RNA samples were quantified using a NanoDrop ND-1000 UV-Vis Spectrophotometer (Palaiseau, France) and the integrity was checked by electrophoresis.

Five micrograms of total RNA samples were reverse-transcribed using the superscript II kit (Invitrogen, Cergy Pontoise, France) and random hexamers (GE Healthcare, Uppsala, Sweden). Real-time PCR was performed using primers reported in Table S1 that also describes the target gene functions. These genes were chosen on the basis of their involvement in the regulation of thyroid hormone and mitochondrial metabolism [18]–[19], their response to heat exposure [19] and their role in the regulation of nutrient utilization and cell defense against oxidative stress [20]–[21]. cDNA samples were subsequently amplified in real time using Sybr Green I Master kit (Roche, Mannheim, Germany) with the LightCycler 480 apparatus (Roche Diagnostics, Meylan, France). A melting curve program was applied from 65 to 95°C in 1 min for each individual sample. Each run included ultrapure water as negative control, samples in triplicate, and control cDNA corresponding to a pool of cDNA from all samples per tissue in duplicate, in addition to the real-time PCR mix.

The relative expression of each target gene was calculated according to the delta-Ct method: Ct (threshold cycle) values for a target gene were normalized to the specimen with the highest expression (minimum Ct value) for that gene, calculated according to the formula: Q = E×(min_Ct_ - sample_Ct_), where Q is the relative Ct value for a given gene, E the PCR efficiency (ranging from 1 to 2 with 100% = 2) calculated from the standard curve, min_Ct_ the minimum Ct value for the gene among all specimens, and sample_Ct_ the Ct value of the gene for the current specimen.

To determine a normalization factor (NF), β-actin, Cytochrome b and 18S were checked for expression stability as non-differentially expressed genes using the geNorm software [22]. Normalized expression (NE) was calculated as the ratio of Q to NF.

### Measurement of levels of β-hydroxyacyl CoA dehydrogenase (HAD), citrate synthase (CS) and lactate dehydrogenase (LDH) activity in PM muscle and liver

HAD, CS and LDH are key enzymes involved in mitochondrial β-oxidation, Krebs cycle and anaerobic glycolysis, respectively. For activity measurement, samples were thawed and homogenized in ice-cold phosphate buffer using an ultra-turrax homogenizer (Ultraturrax, Ilka-Verke, Staufen, Germany). The homogenates were sonicated for 2 minutes at 8.5–8.8 Watts and centrifuged (1,500 *g*, 10 minutes at 4°C) before collecting the supernatant. The activity levels of HAD, CS and LDH were determined at 30°C using the spectrophotometric method of Bass et al. [23].

### Western blotting

Western blotting (WB) was performed on muscle and liver lysates from 34-day-old chickens to analyze the effects of thermal treatments on signaling pathways involved in the regulation of protein translation and of cell stress response. Muscle and liver lysates were prepared as previously described [20]. Protein concentrations were determined using the Bio-Rad protein assay kit (Bio-Rad, USA). Tissue lysates (60 µg protein) were subjected to SDS-PAGE gel electrophoresis and Western blotting using the appropriate antibody. Membranes were also probed with an anti-vinculin antibody to monitor gel loading and to normalize data. After washing, membranes were incubated with an Alexa Fluor secondary antibody (Molecular Probes, Interchim, Montluçon, France). Bands were visualized by Infrared Fluorescence using the Odyssey Imaging System (LI-COR Inc. Biotechnology, Lincoln, NE, USA) and quantified by Odyssey infrared imaging system software (Application software, version 1.2).

### Statistical analysis

All data of mRNA expression, protein expression and enzyme activity were analyzed using the GLM procedure of SAS (SAS Inst. Inc., Cary, NC) with the following model: y_ijk_ = μ+IT_i_+R(IT)_ij_+e_ijk_, where y_ijk_ is the parameter considered for animal k at d 34, μ the general mean, IT_i_ the fixed effect of incubation treatment (i = Control, TM), R(IT)_ij_ the fixed effect of temperature at d 34 during the challenge j nested within incubation treatment i (j = non-challenged or heat-challenged) and e_ijk_ the residual pertaining to animal k. The results are presented as least square means (lsmeans) of main effects: incubation treatment (TM during embryogenesis) and the heat challenge within incubation (nested effect). The gene expression values of PGC-1α, LDHA, avian UCP3, HAD, M-CPT1, COX, IGF-1, MSTN in the muscle and of DIO3 and DIO2 in the liver were log-transformed before being analyzed due to heterogeneity of variance between groups measured with the Levene's test using SAS (SAS Inst. Inc., Cary, NC).

### Expression profile

The MeV software (MultiExperiment Viewer, http://www.tm4.org/mev/) was used to describe the global expression pattern of genes regulating energy metabolism that were differentially expressed as measured by qPCR for at least one of the factors (incubation effect and/or challenge intra incubation effect).

## Results

### Messenger RNA expression of metabolic genes in the PM muscle

**Figure 1:**
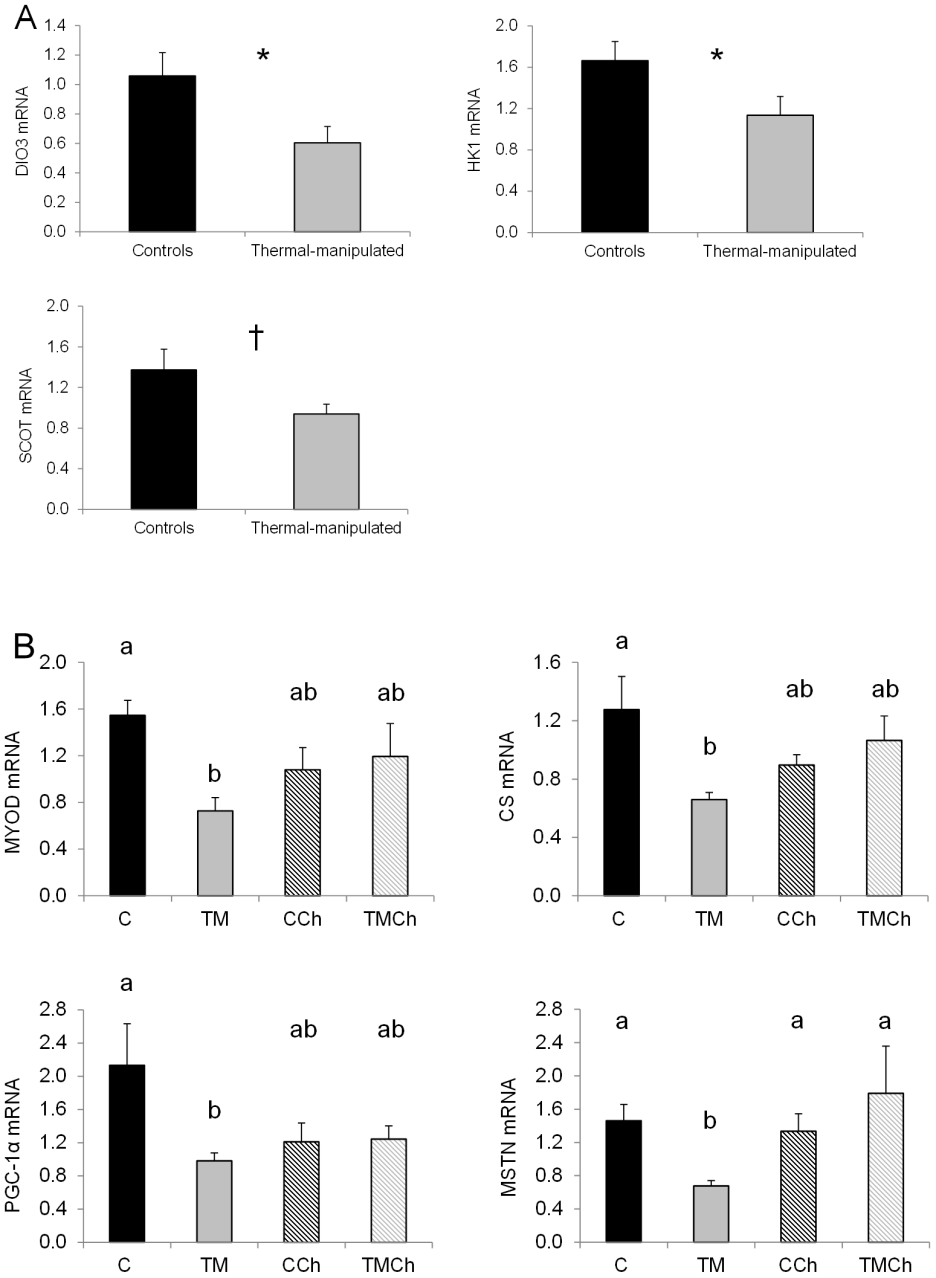
Levels of mRNA of genes affected by thermal manipulation during embryogenesis in the *Pectoralis major* muscle of broiler chickens at d 34. Values were standardized using geNorm factor calculated from the expression of 18S ribosomal RNA, Cytochrome b and β-actin. A) DIO3: deiodinase 3; HK1: hexokinase 1; SCOT: succinyl-CoA: 3-ketoacid CoA transferase; *: *P*<0.05; †: *P*<0.10. B) Chickens were incubated and reared in standard conditions (Controls C), thermally manipulated during embryogenesis and reared in standard conditions (TM), or incubated in standard conditions and exposed to heat challenge at d 34 (CCh) or thermally manipulated during embryogenesis and exposed to heat challenge at d 34 (TMCh). MYOD: myoblast determination protein; GLUT 8: glucose transporter 8; PGC-1α: peroxisome-proliferator-activated receptor (PPAR) γ coactivator 1α; CS: citrate synthase; DIO2: deiodinase 2. Different letters indicate significant differences between treatments (a–b, *P*<0.05) or only a tendency (A–B, *P*<0.10) when both incubation and challenge (incubation) effects or challenge(incubation) effect alone were significant (n = 8 per treatment).

**Figure 2:**
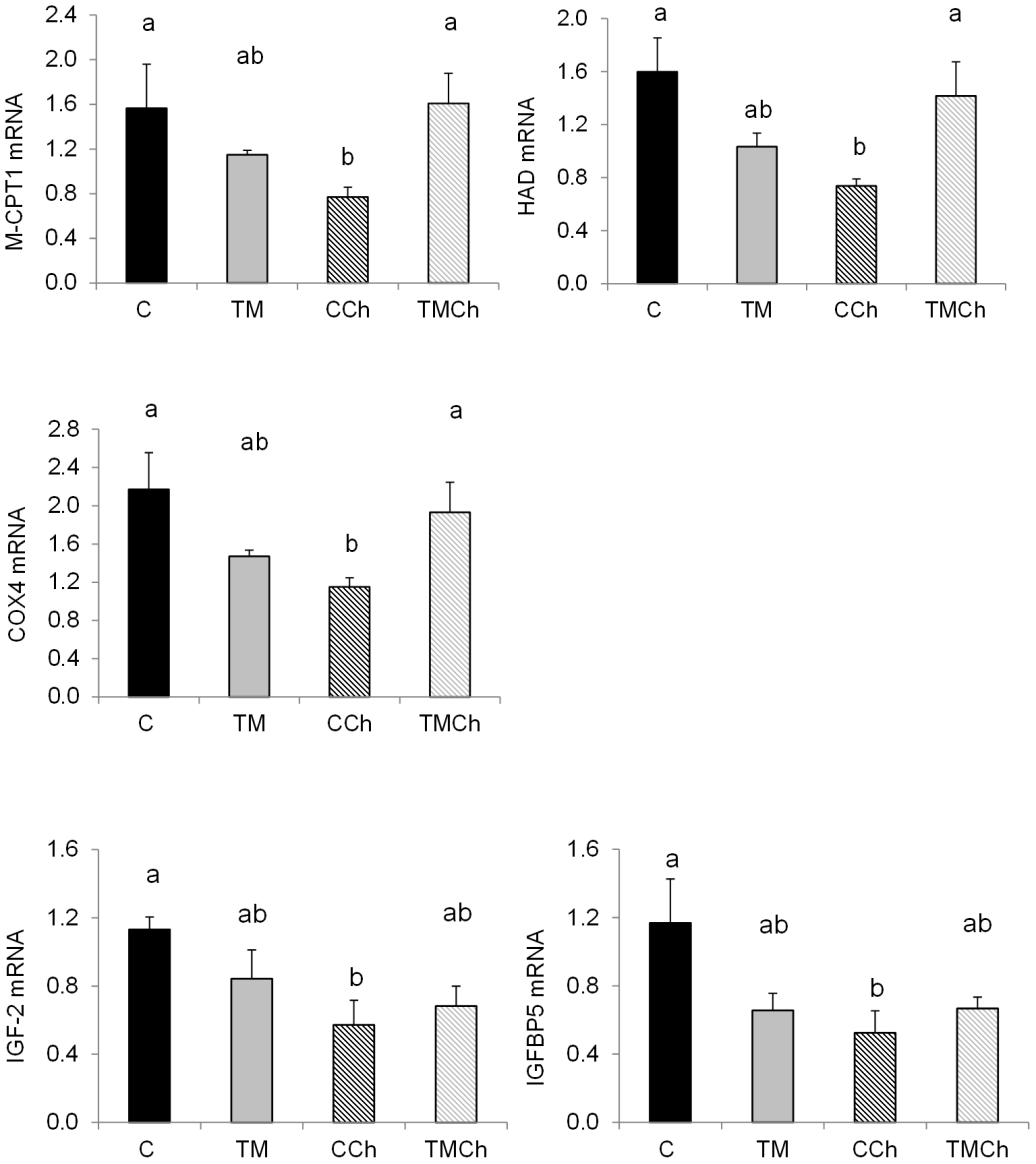
Levels of mRNA of genes affected by heat challenge in the *Pectoralis major* muscle of broiler control chickens at d 34. Chickens were incubated and reared in standard conditions (Controls C), thermally manipulated during embryogenesis and reared in standard conditions (TM), incubated in standard conditions and exposed to heat challenge at d 34 (CCh) or thermally manipulated during embryogenesis and exposed to heat challenge at d 34 (TMCh). Values were standardized using geNorm factor calculated from the levels of expression of 18S ribosomal RNA, Cytochrome b and β-actin. HAD: β -hydroxyacyl-CoA dehydrogenase; COX4: unit 4 of cytochrome c oxidase; IGF-2: insulin growth factor 2; IGFBP5: insulin growth factor binding protein 5. Different letters indicate significant differences between treatments (a–b, *P*<0.05) when both incubation and challenge(incubation) effects or challenge(incubation) effect alone were significant (n = 8 per treatment).

**Figure 3:**
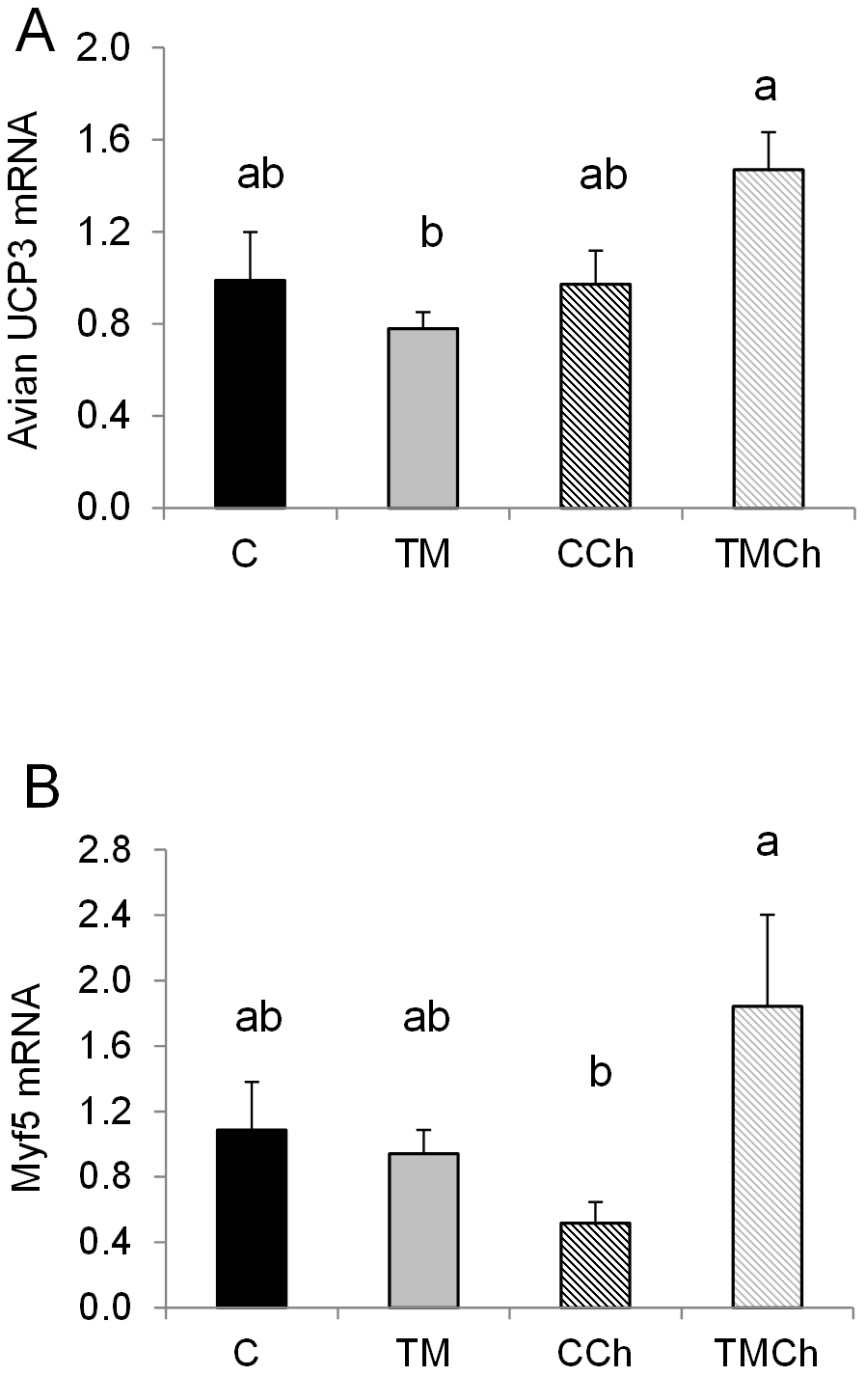
Other patterns of mRNA expression in the *Pectoralis major* muscle of broiler chickens at d 34. Chickens were incubated and reared in standard conditions (Controls C), or thermally manipulated during embryogenesis and reared in standard conditions (TM), incubated in standard conditions and exposed to heat challenge at d 34 (CCh) or thermally manipulated during embryogenesis and exposed to heat challenge at d 34 (TMCh). Values were standardized using geNorm factor calculated from the expression of 18S ribosomal RNA, Cytochrome b and β-actin. A) avian UCP3: avian uncoupling protein; AdMyHC: adult isoform of myosin heavy chain; Atrogin-1. B) Myf5: myogenic factor 5. Different letters indicate significant differences between treatments (a–b, *P*<0.05) or only a tendency (A–B, *P*<0.10) when both incubation and challenge(incubation) effects or challenge(incubation) effect alone were significant (n = 8 per treatment).

Levels of messenger RNA expression in the PM muscle that were significantly affected by TM and/or heat challenge within incubation condition are presented in Figures 1 to 3. The expression of other genes studied that were not significantly affected by treatment is reported in Table S2.

Different expression profiles were observed according to the genes. Expression of the first group of genes (Figure 1A) was lower in TM than in control birds. Indeed, TM during incubation decreased the expression of three genes encoding metabolic enzymes, i.e., deiodinase 3 (DIO3) controlling the local availability of T_3_, hexokinase 1 (HK1) regulating entry into the glycolytic pathway (*P*<0.05), and succinyl-CoA-3-ketoacid CoA transferase (SCOT), involved in the production of ketone bodies from fatty acids (*P* = 0.09, Figure 1A). In the other groups of genes (Figures 1B, 2 and 3) there was an interaction between TM and heat stress at 34 d. In the second group, TM decreased (*P*<0.05) the expression of four genes but only in unchallenged birds (Figure 1B). These genes were myogenic differentiation factor 1 protein (MYOD), citrate synthase (CS), transcription factor peroxisome-proliferator-activated-receptor gamma coactivator 1 alpha (PGC-1α) and myostatin (MSTN). In the third group mRNA expression was decreased following heat challenge but only in the control chickens (C>CCh; Figure 2). They encoded mitochondrial proteins such as muscle isoform of carnitine palmitoyl transferase (M-CPT1) involved in the entry of fatty acids into mitochondria, HAD and the cytochrome oxidase subunit 4 (COX4), but also insulin-like growth factor 2 (IGF-2) and IGF binding protein 5 (IGFBP5) regulating muscle growth. By contrast, the expression of avian uncoupling protein 3 (avian UCP3) involved in the regulation of oxidative stress was increased (*P*<0.05) by heat challenge but only in TM birds (TMCh>TM; Figure 3A). Expression of myogenic transcription factor 5 (Myf5) was significantly higher (*P*<0.05) in the TMCh group than in the CCh group (Figure 3B).

### Messenger RNA expression of metabolic genes in the liver

**Figure 4:**
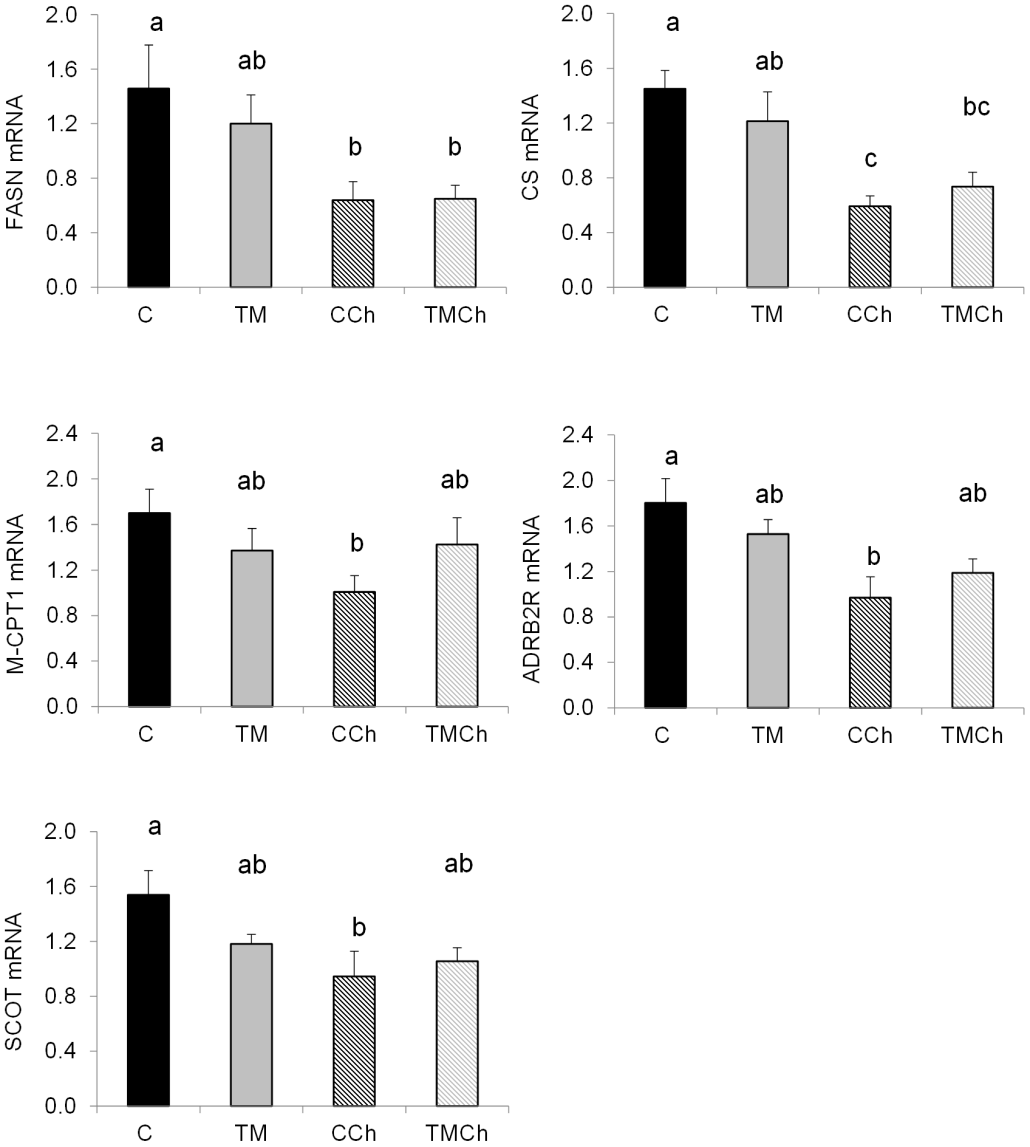
Levels of mRNA of genes affected by heat challenge in the livers of d34 control broiler chickens. Chickens were incubated and reared in standard conditions (Controls C), thermally manipulated during embryogenesis and reared in standard conditions (TM), incubated in standard conditions and exposed to heat challenge at d 34 (CCh), or thermally manipulated during embryogenesis and exposed to heat challenge at d 34 (TMCh). Values were standardized using geNorm factor calculated from the expression of 18S ribosomal RNA, Cytochrome b and β-actin. FASN: fatty acid synthase; CS: citrate synthase; M-CPT1: muscle isoform of carnitine palmitoyltransferase 1; SCOT: succinyl-CoA: 3-ketoacid CoA transferase; ADRB2R: beta-adrenergic receptor 2; HK2: hexokinase 2. Different letters indicate significant differences between treatments (a–b, *P*<0.05) or only a tendency (A–B, *P*<0.10) when both incubation and challenge(incubation) effects or challenge(incubation) effect alone were significant (n = 8 per treatment).

**Figure 5:**
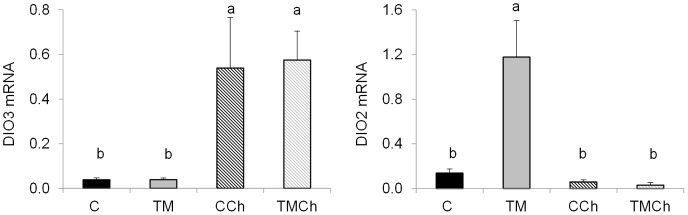
Levels of mRNA of genes altered by heat challenge within incubation treatment in the liver of d 34 broiler chickens. Chickens were incubated and reared in standard conditions (Controls C), thermally manipulated during embryogenesis and reared in standard conditions (TM), incubated in standard conditions and exposed to heat challenge at d 34 (CCh), thermally manipulated during embryogenesis and exposed to heat challenge at d 34 (TMCh). Values were standardized using geNorm factor calculated from the expression of 18S ribosomal RNA, Cytochrome b and β-actin. DIO3: deiodinase 3; DIO2: deiodinase 2; SREBP1: sterol regulatory element binding protein 1. Different letters indicate a tendency to differences between treatments (A–B, *P*<0.10) when both incubation and challenge(incubation) effects or challenge(incubation) effect alone were significant (n = 8 per treatment).

No effect of the incubation treatment alone was observed on the expression of the candidate genes studied in liver tissue. However, the mRNA expression of some genes was affected by heat challenge within incubation conditions, as already observed in PM muscle. The hepatic mRNA expression of 5 genes involved in lipid metabolism was lower (*P*<0.05) in CCh compared to the C group: i.e. fatty acid synthase (FASN) involved in lipogenesis, and CS, M-CPT1, SCOT and β-adrenergic receptor 2 (ADRB2R), all of which are involved in the regulation of fatty acid utilization (Figure 4). However, the expression of these genes was not different between TM and TMCh chickens. The mRNA expression of DIO3 was 25-fold higher (*P*<0.05) in heat-challenged (CCh and TMCh) than in non-challenged (C and TM) birds (Figure 5). Deiodinase DIO2 mRNA expression was increased 10 to 21 fold (*P*<0.05) in TM chickens compared to all other groups (Figure 5). The expression of other candidate genes was not significantly changed in the liver. These are presented in Table S3.

### Enzyme activity

Levels of HAD, LDH and CS activity in the *Pectoralis major* muscle were not affected by incubation or by heat challenge within incubation treatment (Table 1). The levels of activity of HAD and LDH in the liver were not affected by treatment, whereas CS activity was decreased (*P*<0.05) by the incubation treatment in liver tissue (Table 1).

**Table 1:** Effects of thermal manipulation during embryogenesis (TM) and d34 heat challenge on levels of enzyme activity (IU/g protein) in the *Pectoralis major* muscle (PM) and livers of 34-day-old broiler chickens.

		C	TM	CCh	TMCh	*P*-value Incubation effect	*P*-value Challenge (Incubation) effect
PM	HAD	12.30±0.91	13.2±0.83	12.19±0.83	13.53±0.91	0.21	0.96
	LDH	5420±669	5235±605	4932±635	4497±669	0.63	0.63
	CS	21.43±1.03	21.87±0.93	21.92±0.98	20.68±1.03	0.69	0.66
Liver	HAD	60.21±7.28	66.85±6.58	78.08±6.90	68.26±7.28	0.82	0.22
	LDH	72.43±7.83	75.58±7.08	89.34±7.42	78.10±7.83	0.60	0.30
	CS	18.05±0.91	15.96±0.82	19.53±0.86	17.04±0.91	**0.01**	0.35

HAD: β-hydroxyacyl CoA dehydrogenase, CS: citrate synthase and LDH: lactate dehydrogenase activity in PM and Liver. Chickens were incubated and reared in standard conditions (Controls C, n = 9), thermally manipulated during embryogenesis and reared in standard conditions (TM, n = 11), incubated in standard conditions and exposed to heat challenge at 34 d (CCh, n = 10) or thermally manipulated during embryogenesis and exposed to heat challenge at 34d (TMCh, n = 9).

### Kinase phosphorylation in the muscle

**Figure 6:**
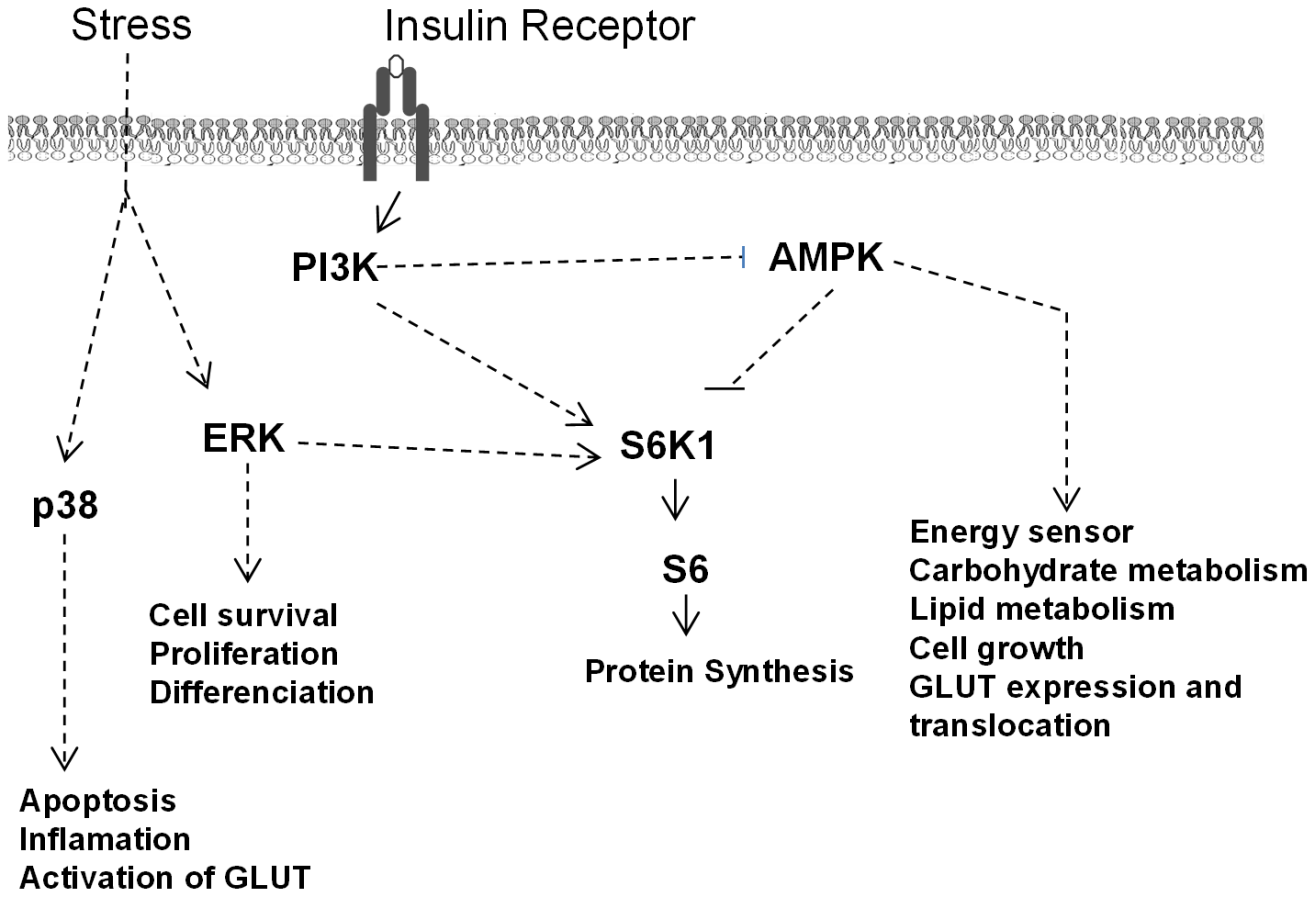
Stress and insulin signaling pathways. ERK: extracellular signal-regulated protein kinase; p38: p38 mitogen-activated protein kinase (p38 MAPK); AMPK: AMP-activated protein kinase; S6K1: p70 S6 kinase or 70 kDA ribosomal protein S6 kinase; S6: ribosomal protein S6. B) Phosphorylation levels of ERK and p38 MAPK (n = 8 per treatment).

**Figure 7:**
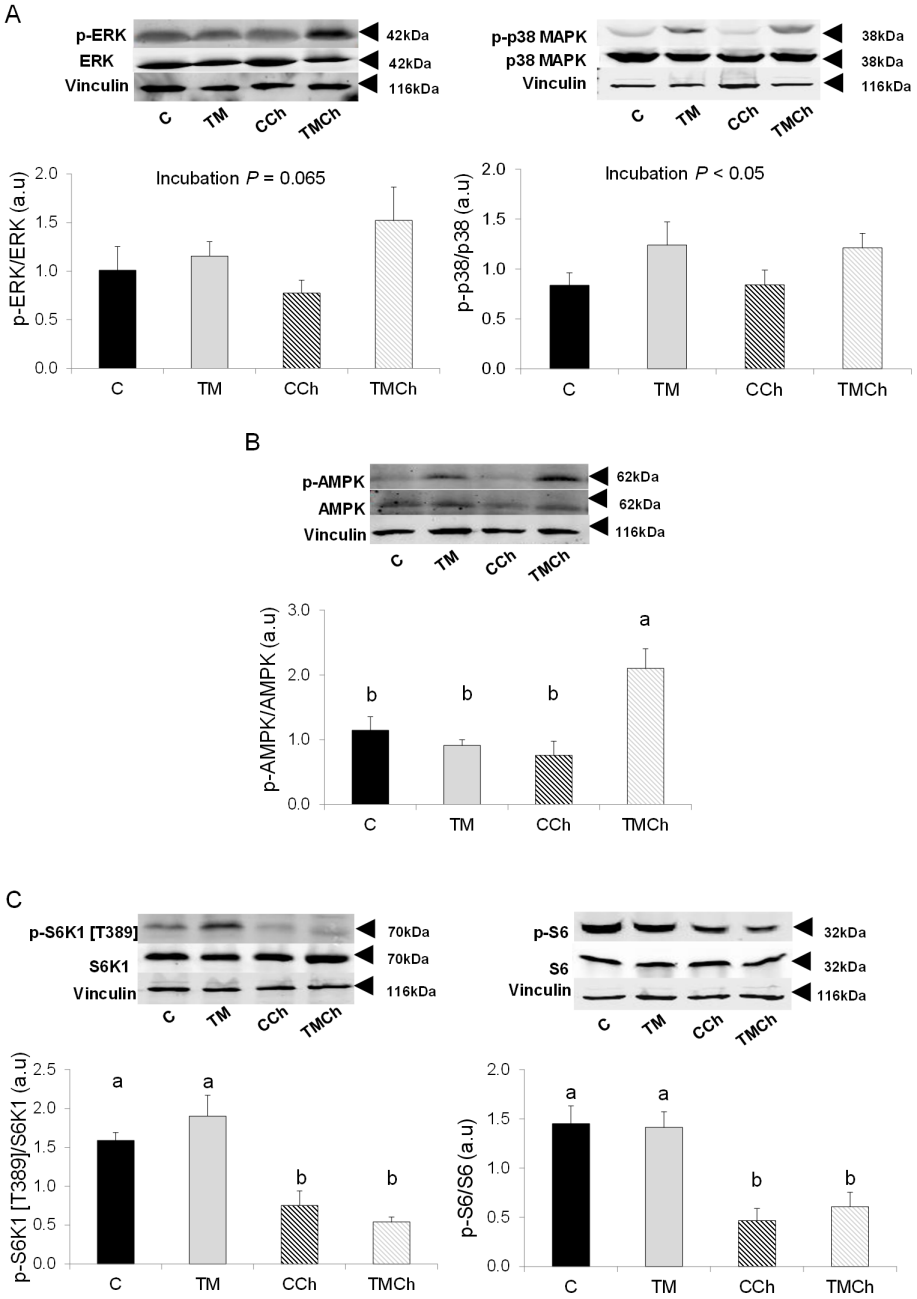
Phosphorylation levels of kinases involved in the regulation of protein and energy metabolism and cellular stress in the *Pectoralis major* muscle. Chickens were incubated and reared in standard conditions (Controls C), thermally manipulated during embryogenesis and reared in standard conditions (TM), incubated in standard conditions and exposed to heat challenge at d 34 (CCh), or thermally manipulated during embryogenesis and exposed to heat challenge at d 34 (TMCh). All western-blots were performed using anti-vinculin antibody as protein loading control. Results are presented as phosphorylated (p-) to total protein ratios. ERK: extracellular signal-regulated protein kinase; p38: p38 mitogen-activated protein kinase (p38 MAPK); AMPK: AMP-activated protein kinase; S6K1: p70 S6 kinase or 70 kDA ribosomal protein S6 kinase; S6: ribosomal protein S6. A) Phosphorylation levels of ERK and p38 MAPK. B) Phosphorylation level of AMPK. C) Phosphorylation levels of S6K1 and S6. Different letters indicate significant differences (*P*<0.05) between treatments (a–b) when both incubation and challenge(incubation) effects or challenge(incubation) effect alone were significant (n = 8 per treatment).

In the PM muscle, phosphorylation of the extracellular signal-regulated protein kinase (ERK) that is involved in cell survival, proliferation, differentiation and in insulin signaling (Figure 6) tended to be greater (*P* = 0.06) in TM than in control birds whether challenged or not (Figure 7A). Similarly, the phosphorylation level of p38 MAP (mitogen-activated protein) kinase, that is involved in apoptosis and cellular stress, was higher (*P*<0.05) in TM than in control birds (Figure 7A). The phosphorylation level of the energy sensor AMP-activated protein kinase (AMPK) was increased (*P*<0.01) in the TMCh group compared to all other groups (Figure 7B). In the signaling pathway regulating protein translation, lower phosphorylation levels of p70S6 kinase (i.e. S6K1) and of ribosomal protein S6 (*P*<0.01) were found after heat challenge for both control and TM birds (Figure 7C).

### Kinase phosphorylation in the liver

**Figure 8:**
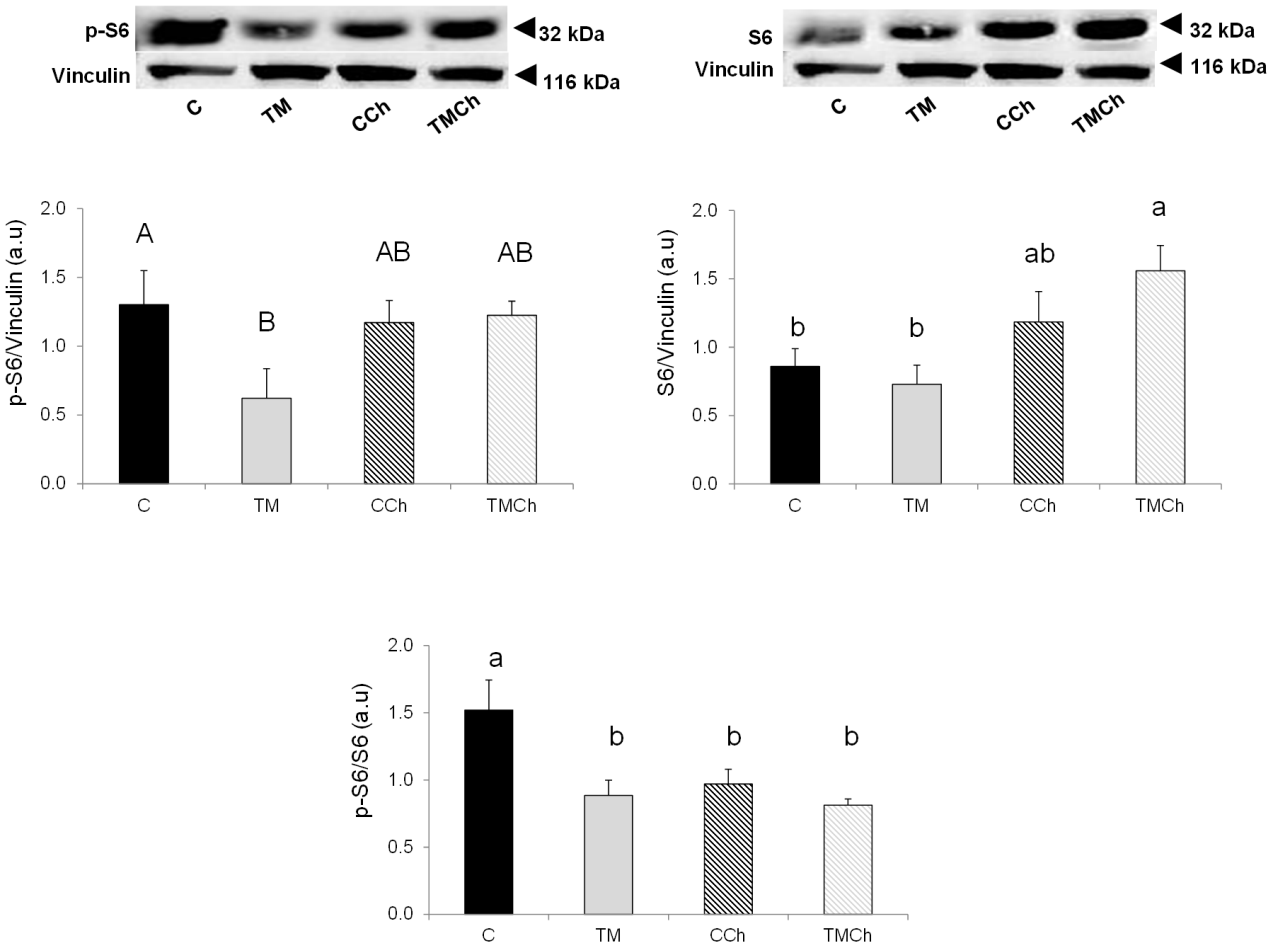
Phosphorylation levels of kinases involved in the regulation of protein metabolism in the liver. Chickens were incubated and reared in standard conditions (Controls C), thermally manipulated during embryogenesis and reared in standard conditions (TM), incubated in standard conditions and exposed to heat challenge at d 34 (CCh), or thermally manipulated during embryogenesis and exposed to heat challenge at d 34 (TMCh). All western-blots were performed using anti-vinculin antibody as protein loading control. Results are presented as phosphorylated (p-) to total protein ratios. S6: ribosomal protein S6. Different letters indicate significant differences between treatments (a–b, *P*<0.05) or only a tendency (A–B, *P*<0.10) when both incubation and challenge(incubation) effects or challenge(incubation) effect alone were significant (n = 8 per treatment).

The p-S6/S6 ratio in the liver was significantly higher in controls than in all other groups (Figure 8; *P*_incubation_<0.01; *P*_challenge(incubation)_<0.05). This was due to both a lower phosphorylation level of p-S6/vinculin in TM than in C (*P*<0.10) and a lower S6/vinculin ratio in C and TM than in TMCh chickens. The phosphorylation levels of other kinases studied in the liver (AMPK, ERK, p38) were not significantly affected, regardless to the nature of the incubation treatment or the challenge within incubation treatment (Table S4).

## Discussion

The aim of this study was to investigate the effects of TM during embryogenesis, combined or not with a subsequent post-hatching heat challenge on candidate genes and pathways controlling body composition, muscle biology, protein and energy metabolism. Previous studies using the same experimental model have provided evidence that the chicken physiology and nutritional partition can be affected by such treatments [6], [9]. Chickens with lowest T_b_ for TM and with highest T_b_ for C were chosen in larger groups in order to better highlight differences between the potentially most and less thermotolerant chickens, respectively. One striking result was that four major patterns of response to thermal treatments were observed among the genes and pathways investigated. First, for a large number of genes or pathways tested there were no significant differences in the responses of chickens to either treatment, or the response to the heat challenge was similar in both TM and control birds. Secondly, mRNA expression or activity for a group of genes or enzymes was clearly reduced by the TM of the embryo, most differences being observed at 21°C, and more rarely at 32°C. This pattern of expression was mainly found in the muscle tissue. A third group comprised genes for which expression was affected by heat challenge in control but not in TM birds, suggesting a specific impact of TM of the embryo on bird's response to heat stress. This pattern of expression occurred in both tissue types. Finally, some genes were affected by the heat challenge only in TM chickens, also suggesting a role of TM in defining the chicken's response to heat challenge.

### Response of TM birds related to the control of body composition and muscle biology

Previous results [8]–[9], [11] have shown positive effects of TM on breast muscle yield and abdominal fat content. We therefore investigated the effects of TM on key genes involved in muscle growth and properties, and in the regulation of body fatness in the present study. Interestingly, under standard conditions at 21°C the muscle mRNA expression of MYOD, a transcription factor involved in the regulation of muscle differentiation [24], decreased in TM chickens. This result suggests that the TM treatment could interfere with the transition between proliferation and differentiation of myoblasts in the muscle. Previous results have shown that TM during late embryogenesis [25] or early post-hatching [26] enhances muscle cell proliferation compared to control conditions and, in the case of posthatch treatment, breast muscle IGF-1 mRNA expression. In our study, Myf 5, that regulates early muscle development events, was not different between TM and control groups when studied under standard rearing conditions (21°C), but was significantly increased following heat challenge in TM birds. The expression of MSTN, a negative regulator of muscle growth, was decreased in the TM group at 21°C, while the mRNA expression of IGF-1, a positive regulator of growth, also tended to decrease in this condition (Table S2). In this study, the heat challenge also affected certain regulators of muscle growth and development, lowering both IGF-2 and IGFBP5 mRNA expression involved in controlling cell survival, differentiation and apoptosis [27], but only in control broilers. These results together suggest that the thermal treatment of embryos may durably affect the regulation of muscle development, which could contribute to the differential breast yields observed in previous studies between TM and control chickens [9], [25]. However, they also indicate possible interference of incubation conditions with the response to subsequent heat challenge, as shown by results concerning the IGF system.

We further studied the regulation of muscle growth and metabolism by measuring the mRNA expression of some key genes involved in proteolysis and by investigating the phosphorylation levels of several kinases regulating protein translation. Indeed, previous studies in our laboratory [19], [28] had provided evidence of the regulation of both protein synthesis and degradation by chronic heat exposure. In our experimental conditions, the thermal manipulation of embryos did not significantly affect regulators of protein degradation and synthesis. However, acute heat exposure at d 34 affected some of the genes and proteins involved in protein turnover. The phosphorylation levels of S6K1 and S6, two kinases regulating protein synthesis [29], were dramatically decreased following heat challenge, suggesting lower stimulation of protein synthesis in challenged birds, as already described in the skeletal muscle of heat-exposed chickens by Temim et al. [28]. A tendency was also observed for atrogin-1, involved in protein degradation, to be upregulated during heat challenge. Incubation conditions thus do not seem to affect protein turnover in the breast muscle of 34-day-old chickens directly nor their response to acute heat stress that did not have different effects on regulators of protein turnover in both the control and TM birds.

As reported by Ain Baziz et al. [30] and Lu et al. [31], carcass adiposity is either unchanged or higher in heat-exposed chickens than in control fast-growing chickens. On the other hand, abdominal fatness is lower in TM than in control broilers [9], [13]. We therefore investigated the regulation of lipogenic and lipolytic pathways in the liver, the main site of lipid synthesis in birds [16]. Surprisingly, there was no effect of the incubation conditions on the hepatic mRNA expression of FASN, a key enzyme controlling hepatic lipogenesis in chickens, or on SREBP-1, M-CPT1, SCOT, HAD or PPARδ that are all involved in fatty acid utilization. This suggests that post-transcriptional regulation of lipogenic enzyme content or activity and blood lipid transfer may explain the decrease in adiposity previously observed in TM compared to control chickens. Regulation of lipid metabolism by heat exposure was observed. Indeed, a 50% decrease in mRNA expression of FASN was induced by heat challenge, suggesting a considerable negative effect on liver lipogenic activity. Concomitantly, the expression of M-CPT1 and SCOT genes, that regulate fatty acid oxidation and ketone body production, respectively, was also decreased by heat challenge, especially in the control group. Therefore, the regulation of both lipogenic and lipolytic pathways appeared to be affected by acute heat stress, although no change in body composition due to heat challenge was observed in our previous study [9].

### Control of heat production and energy metabolism

Thermal manipulation during embryogenesis was reported to decrease body temperature and the plasma triiodothyronine (T_3_) concentration that controls heat production and metabolism [32]–[33], until 28 d in fast-growing chickens [9]. In the present study, a subsample of TM chickens originating from the same study but specifically chosen for low body temperatures did not show lower plasma T_3_ concentrations than controls. Deiodinase DIO2 is involved in the conversion of inactive thyroid hormones T_4_ and reverse T_3_ (rT_3_) into active thyroid hormones T_3_ and diiodothyronine T_2_. Deiodinase DIO3 converts T_3_ into T_2_ and T_4_ into rT_3_ [34]. Our results showed higher DIO3 expression in the liver, the major organ converting plasma T_4_ into T_3_ [34], after heat challenge. This is consistent with the decrease in plasma T_3_ previously reported during heat exposure in birds and mammals [6], [35]. However, the lack of difference between C and TM chickens for DIO3 expression and the specific increase in DIO2 expression in TM birds at 21°C might explain why plasma T_3_ concentrations of TM birds were not lower than those of control birds in the present conditions.

Nevertheless, TM has been shown to decrease O_2_ consumption of embryos, indicating a potentially lower metabolic rate in these animals [11]–[12]. It has also been suggested that TM could affect heat production *via* active local thyroid hormone concentrations. Our study showed that the DIO3 mRNA expression in the breast muscle was significantly decreased by TM in challenged and unchallenged groups, while DIO2 tended to be decreased by TM only in non-challenged chickens (Table S2). These findings suggest that TM has a long-term overall negative effect on thyroid hormone metabolism in the breast muscle. However, the physiological impact of such changes remains to be determined, since the activity of deiodinase enzymes is not necessarily correlated with their mRNA expression.

As a transcriptional gene coactivator, T_3_ binds to its receptor (TR) to interact with thyroid hormone receptor response elements on DNA. A candidate gene for regulating thyroid-stimulated metabolic pathways and mitochondrial biogenesis is transcription factor Peroxisome-Proliferator-Activated-Receptor γ Coactivator 1 α (PGC-1α) [36]–[37]. Indeed, the increase in plasma T_3_ levels and functional maturation of thyroid hormones in chickens coincides with an upregulation of PGC-1α during embryogenesis [38]. It has also been reported that cold exposure upregulates PGC-1α expression in skeletal muscle during establishment of endothermy in birds (around E15; [39]), while one week of chronic heat exposure reduced its mRNA expression in 4-wk-old broiler chickens [19]. In our conditions, PGC-1α was significantly lower in the PM muscle of TM animals than in C, suggesting subsequent modifications in the regulation of genes involved in mitochondrial function. Energy production pathways might thus be modified in the long term by incubation conditions in chickens reared at 21°C, as indicated by decreased levels of gene expression of HK1 (key enzyme in glycolysis), and CS (a key enzyme in the Krebs cycle) in TM, as compared to C chicken muscles. SCOT, involved in the production of ketone bodies, also tended to be affected in muscle by the TM treatment. The heat challenge had no additional effect on the expression of these genes in the muscle of TM animals. These results suggest that the regulation of both mitochondrial metabolism and glycolysis may have been affected in the long term by TM, probably contributing to an overall decrease in energy metabolism in the muscle tissue of TM animals characterized by lower body temperature.

Levels of expression of M-CPT1, HAD and COX4 mRNA were decreased by the heat challenge in the PM muscle of control but not of TM chickens. This indicates that the TM treatment may limit the impact of the heat challenge on the expression of these genes involved in the regulation of the β-oxidation of lipids and of the respiratory chain, respectively [18]. Although Azad et al. [40] had previously reported decreased HAD activity in the muscle of chickens exposed to 34°C for 15 d we did not observe any effect of TM or heat challenge on the activity of enzymes involved in muscle energy metabolism in the present study, probably due to the lower intensity and shorter duration of the thermal exposure applied.

In order to obtain an overall picture of the expression pattern of target genes regulating energy metabolism in the PM muscle (controlling mitochondrial metabolism, fatty acid utilization or glycolytic metabolism), we represented differentially expressed genes for at least one factor (incubation and/or challenge intra incubation condition) on the same Figure. Our results showed that expression of genes regulating energy metabolism tended overall to be lower in TM, TMCh and CCh chickens than in controls (Figure S1). Metabolic heat production may thus be as reduced in TM broilers as in heat-challenged animals. This suggests that effective thermal manipulation inducing low T_b_ may already prepare animals to tolerate high ambient temperatures by downregulating key genes involved in energy production pathways, meaning that subsequent acute heat exposure induces no or only slight further modification of the expression of these genes.

The changes in mRNA expression observed in the muscle in response to TM were concomitant with modifications of the activation of AMPK involved in energy sensing. Indeed, in the present study, we found an increase in AMPK phosphorylation in TMCh chickens as compared to all other groups. AMPK has been shown to trigger skeletal muscle glucose utilization in chicken embryos [41]. In mammals activation of AMPK induces the membrane translocation of the GLUT transporter in skeletal muscle [42]. We previously demonstrated that the regulation of glucose utilization was modulated by combined embryo and postnatal thermal treatment, with higher plasma glucose concentrations in TMCh chickens than in all other groups, despite unchanged plasma insulin concentrations in TMCh and CCh chickens [9]. The higher phosphorylation level of AMPK in TMCh chickens might thus represent a signal inducing the transport of glucose to the skeletal muscle *via* translocation of the glucose transporter in response to high glycaemia. This might also be the result of an increased need for energy production pathways in response to the acute heat challenge in TM chickens characterized in standard conditions by down-regulated ATP-generating pathways as suggested by lower levels of PGC-1α mRNA expression.

In order to characterize the metabolic changes induced by our treatments in major organs regulating body composition and animal metabolism, we also focused on target pathways controlling hepatic lipogenesis and energy utilization. In the liver, citrate synthase activity was lower in TM than in C animals, possibly reflecting lower intensity of energy transfer in mitochondria affected by the embryo treatment, and consistent with an overall decrease in metabolic intensity in TM birds. This effect was however not the same as that observed at the mRNA level, where the expression of CS was lower at 32°C than at 21°C only in control chickens. Moreover, gene expression in the muscle and in the liver was affected differently by treatments, and only DIO2 mRNA expression in the latter was changed by TM. In accordance with previous results [43]–[44] showing hepatic metabolic modifications during heat exposure, lower levels of mRNA expression of SCOT, M-CPT1, the β-adrenergic receptor ADRB2R and of HK2, were found in the liver following heat challenge. However, these lower expression levels were mainly observed in control birds, and were intermediate in TM chickens. This might reflect a possible limitation of the heat challenge effect on fatty acid mitochondrial utilization, production of ketone bodies, response to β-adrenergic pathway and glycolysis, in the livers of TM chickens compared to control chickens.

### Mechanisms involved in the stress responses of chickens

Thermal manipulation during incubation induced specific effects on mechanisms controlling stress responses and apoptosis in the PM muscle, but not in the liver. One of these was the MAP kinase signaling pathway. It was recently shown that the activation of p38 MAPK is induced by oxidative stress and that its upregulation is responsible for the downregulation of generation of free radicals and for *in vitro* survival of mammalian cell lines [45]. The upregulation of p38 MAPK observed in TM chickens and, to a lesser extent, of the MAP kinase ERK also involved in cell stress response, may represent an adaptive mechanism for regulating oxidative stress and cell survival in both thermoneutral and heat challenge conditions. UCP is also known to control oxidative stress. Expression of avian UCP3 mRNA was upregulated in the muscle of TMCh compared to TM birds. Avian UCP3 has previously been shown to be affected by nutrition [46], genotype [18], [47], and ambient temperature [48]–[50], and its expression is regulated by the thyroid axis and the beta-adrenergic pathway [20]–[21], [51]. It is thought to protect muscle tissue from oxidative injury by reducing oxidative stress that is particularly increased during acute heat exposure [52]. The overexpression of avian UCP3 in the muscle during heat challenge in TM animals may thus contribute to protection against oxidative stress, whereas this pathway seemed not to be affected in heat-challenged control chickens. In addition to these different responses to heat-induced oxidative stress, we have previously reported potentially lower stress responses in TM animals during heat challenge, as indicated by modified plasma corticosterone concentration and blood heterophil to lymphocyte ratio [9], a well-known marker of the stress response in avian species [53].

To conclude, chickens submitted to TM during embryogenesis and characterized by low T_b_ exhibited long-term modifications of their metabolism. TM may contribute to a decrease in the intensity of energy metabolism in the liver and breast muscle, potentially resulting in lower heat production, or may mitigate the effects of heat stress later in life. We also report modifications of pathways regulating muscle cell stress responses and development that may contribute to the greater tolerance of thermal-manipulated chickens subsequently exposed to heat stress. These potentially “programing” effects of thermal manipulation of the embryo may be partly due to epigenetic regulation that has already been suggested to be involved in the modification of gene expression in the case of early post-hatch thermal exposure. Whether such mechanisms are involved in the regulation observed in the present study remains to be elucidated.

## Supporting Information

Figure S1 **Expression profiles of target genes involved in energy metabolism and differentially expressed in at least one condition (whether incubation treatment and/or heat challenge (intra incubation)).** Genes included were peroxisome proliferator activated receptor coactivator 1 alpha, citrate synthase, glucose transporter 8, hexokinase 1, succinyl-CoA: 3-ketoacid CoA transferase, cytochrome oxidase subunit 4, β-hydroxyl-acyl CoA dehydrogenase, muscle isoform of carnitine palmitoyl transferase 1 with the average expression of these genes in red. Blue dashes correspond to the highest or lowest points of the average line (n = 8 per treatment).
(TIF)

Table S1 **Primers used for qRT-PCR.**
(DOCX)

Table S2 **Levels of m-RNA expression in the** ***Pectoralis major*** **muscle of 34-day-old broiler chickens.**
(DOCX)

Table S3 **Levels of m-RNA expression in the livers of 34-day-old broiler chickens.**
(DOCX)

Table S4 **Levels of phosphorylation of kinases in the livers of 34-day-old broiler chickens.**
(DOCX)

File S1 **Individual data of mRNA expressions, phosphorylation levels of kinases and metabolic enzyme activities in the** ***Pectoralis major*** **muscle and in the livers of 34-day-old broiler chickens.**
(XLS)
