# Supplementary figures and images for: Thermal Manipulation during Embryogenesis Has Long-Term Effects on Muscle and Liver Metabolism in Fast-Growing Chickens
Source: PLoS One. 2014 Sep 2;9(9):e105339. doi: 10.1371/journal.pone.0105339 (PMC4152147; doi:10.1371/journal.pone.0105339)

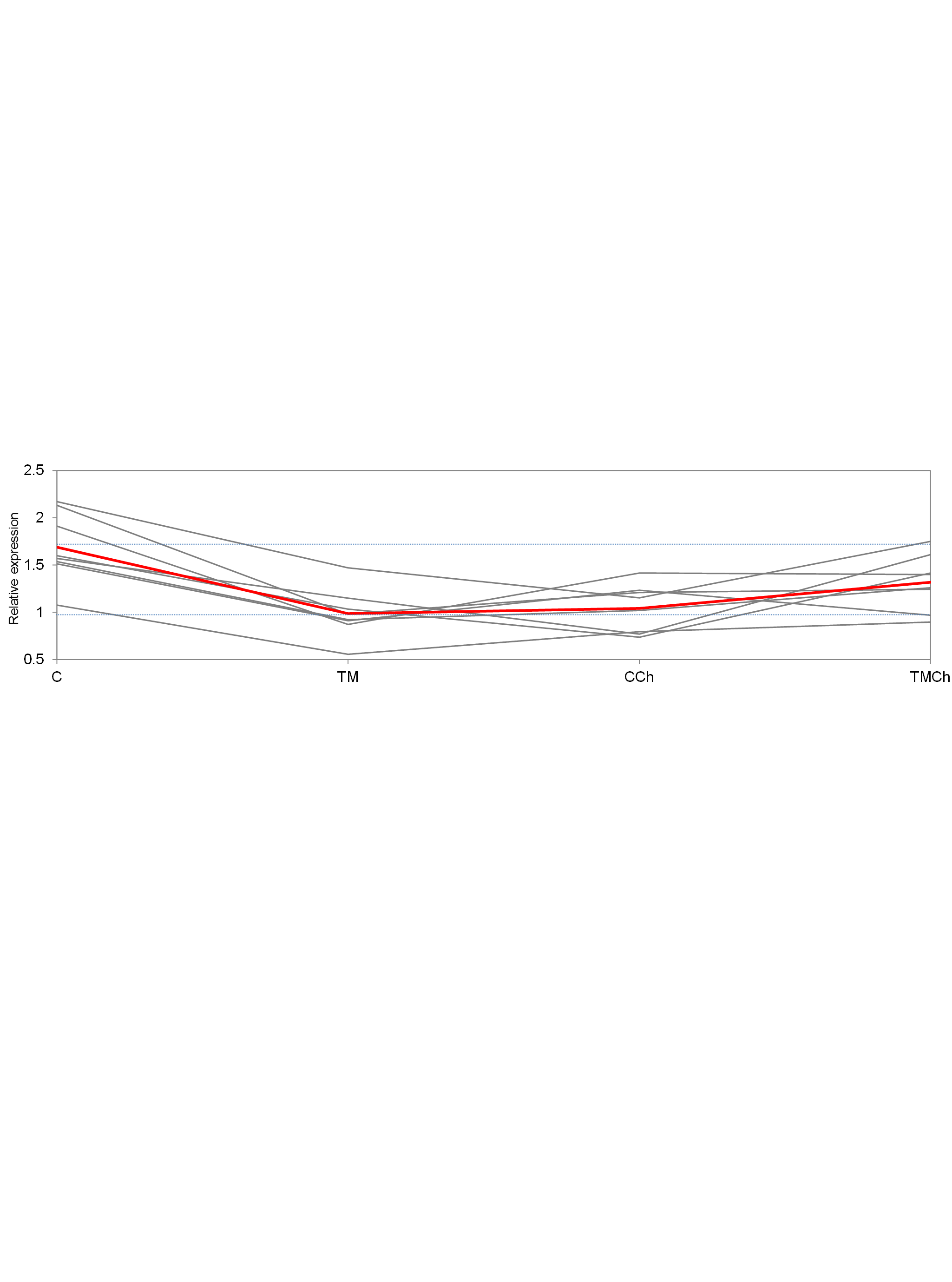

Supplement: Figure S1 — Expression profiles of target genes involved in energy metabolism and differentially expressed in at least one condition (whether incubation treatment and/or heat challenge (intra incubation)). Genes included were peroxisome proliferator activated receptor coactivator 1 alpha, citrate synthase, glucose transporter 8, hexokinase 1, succinyl-CoA: 3-ketoacid CoA transferase, cytochrome oxidase subunit 4, β-hydroxyl-acyl CoA dehydrogenase, muscle isoform of carnitine palmitoyl transferase 1 with the average expression of these genes in red. Blue dashes correspond to the highest or lowest points of the average line (n = 8 per treatment). (TIF) [file pone.0105339.s001.tif]
