# Supplementary material for: Thermal Manipulation during Embryogenesis Has Long-Term Effects on Muscle and Liver Metabolism in Fast-Growing Chickens
Source: PLoS One. 2014 Sep 2;9(9):e105339. doi: 10.1371/journal.pone.0105339 (PMC4152147; doi:10.1371/journal.pone.0105339)
Supplement: Table S1 — Primers used for qRT-PCR. (DOCX) [file pone.0105339.s002.docx]

| **Table S1. Primers used for qRT-PCR.** | | | |
| --- | --- | --- | --- |
| \| \| **Genes** \| \| **Primers** \| **Accession Number** \| \| \| **Function** \| \| \| \| --- \| --- \| --- \| --- \| --- \| --- \| --- \| --- \| --- \| \| ***Normalization genes*** \| \| \| \| \| \| \| \| \| \| 18S \| Fw: TCCAGCTAGGAATAATGGAATAGGA \| \| HQ873432.1 \| \| Housekeeping genes \| \| \| \| \|  \| Rev: CCGGCCGTCCCTCTTAAT \| \| \| β-actin \| Fw: CTGGCACCTAGCACAATGAA \| \| NM 205518.1 \| \| Housekeeping genes \| \| \| \| \|  \| Rev: CTGCTTGCTGATCCACATCT \| \| \| cytb \| Fw: CGGACGAGGCCTATACTACG \| \| NC_001323 \| \| Housekeeping genes \| \| \| \| \|  \| Rev: GGGAGAACATAGCCCACAAA \| \| \| ***Thyroid hormone metabolism*** \| \| \|  \| \|  \| \| \| \| \| DIO1 \| Fw: TTTCAGCTTCCAGTATTTGCTCTTT \| \| NM 001097614.1 \| \| Peripheral deiodination of thyroid hormones \| \| \| \| \|  \| Rev: GTCCCTCGTGGGCTTCGT \| \| \| DIO2 \| Fw: AGAAGCTCCGAACTCCAGTGTAA \| \| NM 204114.3 \| \| Peripheral deiodination of thyroid hormones \| \| \| \| \|  \| Rev: CCCCGGCTCCTTCAAAA \| \| \| DIO3 \| Fw: GGAAATCGTGTCTGATGTTGTCA \| \| NM 001122648.1 \| \| Peripheral deiodination of thyroid hormones \| \| \| \| \|  \| Rev: AACCCCCCCCATAAATAGGA \| \| \| ***Energy metabolism*** \| \| \|  \| \|  \| \| \| \| \| PGC-1α \| Fw: GGGACCGGTTTGAAGTTTTTG \| \| NM 001006457.1 \| \| \| Transcriptional co-activator involved in energy metabolism and activation of mitochondrial biogenesis \| \| \| \|  \| Rev: GGCTCGTTTGACCTGCGTAA \| \| \| PPARα \| Fw: CAAACCAACCATCCTGACGAT \| \| NM 001001464.1 \| \| \| Transcription factor involved  in activation of lipid metabolism \| \| \| \|  \| Rev: GGAGGTCAGCCATTTTTTGGA \| \| \| PPARδ \| Fw: CATGGAGCCCAAGTTTGAGT \| \| NM 204728.1 \| \| \| Transcription factor involved in  activation of lipid metabolism \| \| \| \|  \| Rev: CGGAGGATGTTGTCTTGGAT \| \| \| HAD \| Fw: ATCCTTGCAAATCACGCAGTT \| \| NM 001277897.1 \| \| \| Mitochondrial beta-oxidation  of short chain fatty acid \| \| \| \|  \| Rev: AATGGAGGCCACCAAATCG \| \| \| CS \| Fw: AGGGATTCCATCTGGAACACACT \| \| XM 003643956.2 \| \| \| Krebs cycle \| \| \| \|  \| Rev: CAGCGTGTAGTACTTCATCTCCT \| \| \| COX4 \| Fw: CTTTCCACCTCCATCTGTGTGA \| \| NM 001030577.1 \| \| \| Oxidative Phosphorylation \| \| \| \|  \| Rev: TGCTGGATGGCTGAAATCG \| \| \| HK1 \| Fw: CGTTGTGAAGTTGGCTTGATC \| \| NM 204101.1 \| \| \| Glycolysis \| \| \| \|  \| Rev: CCACGGTCGATCTCTCTATCA \| \| \| HK2 \| Fw: GTCTGCGCCGCATCAA \| \| NM 204212.1 \| \| \| Glycolysis \| \| \| \|  \| Rev: CTTCCTCACTGTCTTATGGAGGC \| \| \| GCK \| Fw: TGCTGCGGCTGGTGGATGAGAA \| \| XM 004949994.1 \| \| \| Glycolysis \| \| \| \|  \| Rev: ATCTGCTTGCGGTCTCCTGA \| \| \| GLUT8 \| Fw: CTGGAGGAATACTGGGAGGC \| \| NM 204375.1 \| \| \| Glucose Transporter \| \| \| \|  \| Rev: CACCACCATCAACTGGACAA \| \| \| LDHA \| Fw: TTAACTTGGTCCAACGCAACGTCAAT \| \| NM 205284.1 \| \| \| Conversion of pyruvate into lactate \| \| \| \|  \| Rev: TCCACTGGGTTTGAGACAATCAG \| \| \| ANT \| Fw: TATCAGCTGGATGATTGCACAGA \| \| AB088686.1 \| \| \| Mitochondrial ADP/ATP translocator \| \| \| \|  \| Rev: ACATGATATCAGCTCCTTTGCGT \| \| \| M-CPT1 \| Fw: GATTTCTGCTGCTTCCAATTCG \| \| DQ314726.1 \| \| \| Rate limiting enzyme of  fatty acid oxidation (muscle) \| \| \| \|  \| Rev: TGCAGCGCGATCTGAATG \| \| \| L-CPT1 \| Fw: GCCCTGATGCCTTCATTCAA \| \| AY675193 \| \| \| Rate limiting enzyme of fatty  acid oxidation (liver) \| \| \| \|  \| Rev: ATTTTCCCATGTCTCGGTGA \| \| \| FASN \| Fw: TCTCGATCTGGCATACGAACTG \| \| NM 205155.2 \| \| \| Formation of long-chain-fatty acids \| \| \| \|  \| Rev: CAACTGGCTCGAGCTTCAAAG \| \| \| SREBP1 \| Fw: GTCGGCGATCCTGAGGAA \| \| NM 204126.1 \| \| \| Transcriptional activator required for lipid homeostasis \| \| \| \|  \| Rev: CTCTTCTGCACGGCCATCTT \| \|  \| \| \| \| SCOT \| Fw: CGCCAGTATTTATCTGGTGAGCTT \| \| NM 001006578.2 \| \| \| Ketone body catabolism \| \| \| \|  \| Rev: CTGCTCCTCCTGCTGTAATACGTT \| \| \| ADRB2R \| Fw: ACCTGCCTCTTGTGGTCATGGT \| \| XM 004950587.1 \| \| \| β2 Adrenergic receptor \| \| \| \|  \| Rev: GGATGTGAAACCTCCCCTCACT \| \| \| **Genes** \| **Primers** \| \| \| Accession Number \| \| \| \| Function \| \| ***Protein metabolism*** \| \| \| \|  \| \| \| \|  \| \| Atrogin-1 \| Fw: GACGCGCTTTCTCGATGAG \| \| NM 001030956.1 \| \| \| \| Regulation of proteolysis \| \| \|  \| Rev: CCTTGTTATTCAGTAGGTCTTTTTTCCT \| \| \| MuRF \| Fw: TGTCTATGGGCTGCAGAGGAA \| \| BM488754.1 \| \| \| \| Regulation of proteolysis \| \| \|  \| Rev: GGTGCTCCCCCTTCTTGAGT \| \| \| ***Muscle growth and development*** \| \| \|  \| \| \| \|  \| \| \| IGF-1 \| Fw: TCAACAGTCTTTCAACACAATTAGTTAAGT \| \| KC242240.1 \| \| \| \| Regulator of skeletal muscle growth \| \| \|  \| Rev: ACACAGGCCAAGGTAGAAGAAATG \| \| \| IGF-2 \| Fw: TGTGCCAAGTCCGTCAAGTC \| \| NM 001030342.1 \| \| \| \| Regulator of skeletal muscle growth \| \| \|  \| Rev: TTGGCATGAGATGGCTTCTG \| \| \| IGFBP5 \| Fw: CAAGCCAAGGCCGAACGGGA \| \| XM 422069.4 \| \| \| \| Affects interaction of IGFs with cell surface \| \| \|  \| Rev: TCCCCGGCAGCTTCATGCCA \| \| \| MSTN \| Fw: AGCGGGTAGCGACAACATC \| \| GU181328.1 \| \| \| \| Regulator of skeletal muscle growth \| \| \|  \| Rev: GCTTTTGATGAGACTGGACGAG \| \| \| MYOD \| Fw: GGAATCACCAAATGACCCAAAG \| \| FJ977569.1 \| \| \| \| Myogenic regulation factor \| \| \|  \| Rev: TCACTCAGGTTTCCTCCTTCCT \| \| \| Myf5 \| Fw: ACTCCCCAAAGTGGAGATCCTG \| \| NM 001030363.1 \| \| \| \| Myogenic regulation factor \| \| \|  \| Rev: CAGTCCGCCATCACATCG \| \| \| Myogenin \| Fw: CGGAGGCTGAAGAAGGTGAAC \| \| NM 204184.1 \| \| \| \| Myogenic regulation factor \| \| \|  \| Rev: AGGCGCTCGATGTACTGGAT \| \| \| AdMyHC \| Fw: CCAAATTCCGCAAGATCCAAC \| \| NM 001013397.2 \| \| \| \| Global muscle contraction \| \| \|  \| Rev: CTTATGCCACTTTGTTGTCACGAC \| \| \| EmbMyHC \| Fw: AGGAGCTGTCCAATGTCAACCTC \| \| XM 415578.4 \| \| \| \| Global muscle contraction \| \| \|  \| Rev: GCAGAAGAAAGCAACAGAGGGTTC \| \| \| SlowMyHC \| Fw: TTGAAGGTGATGACCTCCTTGG \| \| NM 204587.1 \| \| \| \| Global muscle contraction \| \| \|  \| Rev: CAACCTGGTGAAGTACCGCAAG \| \| \| NeoMyHC \| Fw: TGCCTCAGGTCACACTTTAGC \| \| XM 001231408.3 \| \| \| \| Global muscle contraction \| \| \|  \| Rev: AGCTGTCCAATGTCAACCTTTCC \| \| \| PAX7 \| Fw: CAACTCGCAGCATTCAACCAT \| \| DQ471304.1 \| \| \| \| Myogenic differentiation factor \| \| \|  \| Rev: ACGGTGCTGCCTCCATCTT \| \| \| ***Stress and Oxidative stress*** \| \| \| \|  \| \| \| \|  \| \| NFκB \| Fw: CGGATCCGCACCAATAACA \| \| [NM 205129.1](http://www.ncbi.nlm.nih.gov/nucleotide/45384099?report=genbank&log$=nucltop&blast_rank=1&RID=E4D1HNR8014) \| \| \| \| Transcription factor involved in inflammation, immunity, differentiation,  cell growth, tumorigenesis and apoptosis \| \| \|  \| Rev: CGTTCACCCACACCTGGAA \| \| \| SOD3 \| Fw: CCAGTGATGGCTGATAATGAGACT \| \| XM 420760.4 \| \| \| \| Antioxidant enzyme \| \| \|  \| Rev: CTATTTTGGAGCTGGGCTTCA \| \| \| avian UCP3 \| Fw: CTACGACCTCATCAAGGACACA \| \| AB088685.1 \| \| \| \| Limitation of oxidative stress;  mitochondrial transporter \| \| \|  \| Rev: GAAGGCAGCCACGAAGTGA \| \| \|  \| Accession Number \| Function \| \| --- \| --- \| --- \| --- \| --- \| --- \| --- \| --- \| --- \| --- \| --- \| --- \| --- \| --- \| --- \| --- \| --- \| --- \| --- \| --- \| --- \| --- \| --- \| --- \| --- \| --- \| --- \| --- \| --- \| --- \| --- \| --- \| --- \| --- \| --- \| --- \| --- \| --- \| --- \| --- \| --- \| --- \| --- \| --- \| --- \| --- \| --- \| --- \| --- \| --- \| --- \| --- \| --- \| --- \| --- \| --- \| --- \| --- \| --- \| --- \| --- \| --- \| --- \| --- \| --- \| --- \| --- \| --- \| --- \| --- \| --- \| --- \| --- \| --- \| --- \| --- \| --- \| --- \| --- \| --- \| --- \| --- \| --- \| --- \| --- \| --- \| --- \| --- \| --- \| --- \| --- \| --- \| --- \| --- \| --- \| --- \| --- \| --- \| --- \| --- \| --- \| --- \| --- \| --- \| --- \| --- \| --- \| --- \| --- \| --- \| --- \| --- \| --- \| --- \| --- \| --- \| --- \| --- \| --- \| --- \| --- \| --- \| --- \| --- \| --- \| --- \| --- \| --- \| --- \| --- \| --- \| --- \| --- \| --- \| --- \| --- \| --- \| --- \| --- \| --- \| --- \| --- \| --- \| --- \| --- \| --- \| --- \| --- \| --- \| --- \| --- \| --- \| --- \| --- \| --- \| --- \| --- \| --- \| --- \| --- \| --- \| --- \| --- \| --- \| --- \| --- \| --- \| --- \| --- \| --- \| --- \| --- \| --- \| --- \| --- \| --- \| --- \| --- \| --- \| --- \| --- \| --- \| --- \| --- \| --- \| --- \| --- \| --- \| --- \| --- \| --- \| --- \| --- \| --- \| --- \| --- \| --- \| --- \| --- \| --- \| --- \| --- \| --- \| --- \| --- \| --- \| --- \| --- \| --- \| --- \| --- \| --- \| --- \| --- \| --- \| --- \| --- \| --- \| --- \| --- \| --- \| --- \| --- \| --- \| --- \| --- \| --- \| --- \| --- \| --- \| --- \| --- \| --- \| --- \| --- \| --- \| --- \| --- \| --- \| --- \| --- \| --- \| --- \| --- \| --- \| --- \| --- \| --- \| --- \| --- \| --- \| --- \| --- \| --- \| --- \| --- \| --- \| --- \| --- \| --- \| --- \| --- \| --- \| --- \| --- \| --- \| --- \| --- \| --- \| --- \| --- \| --- \| --- \| --- \| --- \| --- \| --- \| --- \| --- \| --- \| --- \| --- \| --- \| --- \| --- \| --- \| --- \| --- \| --- \| --- \| --- \| --- \| --- \| --- \| --- \| --- \| --- \| --- \| --- \| --- \| --- \| --- \| --- \| --- \| --- \| --- \| --- \| --- \| --- \| --- \| --- \| --- \| --- \| --- \| --- \| --- \| --- \| --- \| --- \| --- \| --- \| --- \| --- \| --- \| --- \| --- \| --- \| --- \| --- \| --- \| --- \| --- \| --- \| --- \| --- \| --- \| --- \| --- \| --- \| --- \| --- \| --- \| --- \| --- \| --- \| --- \| --- \| --- \| --- \| --- \| --- \| --- \| --- \| --- \| --- \| --- \| --- \| --- \| --- \| --- \| --- \| --- \| --- \| --- \| --- \| --- \| --- \| --- \| --- \| --- \| --- \| --- \| --- \| --- \| --- \| --- \| --- \| --- \| --- \| --- \| --- \| --- \| --- \| --- \| --- \| --- \| --- \| --- \| --- \| --- \| --- \| --- \| --- \| --- \| --- \| --- \| --- \| --- \| --- \| --- \| --- \| --- \| --- \| --- \| --- \| --- \| --- \| --- \| --- \| --- \| --- \| --- \| --- \| --- \| --- \| --- \| --- \| --- \| --- \| --- \| --- \| --- \| --- \| --- \| --- \| --- \| --- \| --- \| --- \| --- \| --- \| --- \| --- \| --- \| --- \| --- \| --- \| --- \| --- \| --- \| --- \| --- \| --- \| --- \| --- \| --- \| --- \| --- \| --- \| --- \| --- \| --- \| --- \| --- \| --- \| --- \| --- \| --- \| --- \| --- \| --- \| --- \| --- \| --- \| --- \| --- \| --- \| --- \| --- \| --- \| --- \| --- \| --- \| --- \| --- \| --- \| --- \| --- \| --- \| --- \| --- \| --- \| --- \| --- \| --- \| --- \| --- \| --- \| --- \| --- \| --- \| --- \| --- \| --- \| --- \| --- \| --- \| --- \| --- \| --- \| --- \| --- \| --- \| --- \| --- \| --- \| --- \| --- \| --- \| --- \| --- \| --- \| --- \| --- \| --- \| --- \| --- \| --- \| --- \| --- \| --- \| --- \| --- \| --- \| --- \| --- \| --- \| --- \| --- \| --- \| --- \| --- \| --- \| --- \| --- \| --- \| --- \| --- \| --- \| --- \| --- \| --- \| --- \| --- \| --- \| --- \| --- \| --- \| --- \| --- \| --- \| --- \| --- \| --- \| --- \| --- \| --- \| --- \| --- \| --- \| --- \| --- \| --- \| --- \| --- \| --- \| --- \| --- \| --- \| --- \| --- \|   **Gene** | **Primers** | **Gene** | **Primers** |

18S: ARNr 18S; cytb: cytochrome b; CS: citrate synthase; DIO1: deiodinase 1; DIO2: deiodinase 2; DIO3: deiodinase 3; PGC-1α: peroxisome proliferator-activated receptor coactivator-1α; PPAR: peroxisome proliferator-activated receptor; HAD: β-hydroxyacyl-CoA dehydrogenase; CS: citrate synthase; COX4: unit 4 of cytochrome c oxidase; HK1: hexokinase 1; HK2: hexokinase 2; GCK: glucokinase; GLUT 8: glucose transporter 8; LDHA: lactate dehydrogenase; ANT: adenine nucleotide translocase; M-CPT1: muscle carnitine palmitoyltransferase 1; L-CPT1: liver carnitine palmitoyltransferase 1; FASN: fatty acid synthase; SREBP1: sterol regulatory element binding protein 1; SCOT: succinyl-CoA: 3-ketoacid CoA transferase; ADRB2R: beta-adrenergic receptor 2; MuRF: muscle specific ubiquitin ligase; IGF-1: insulin growth factor 1; IGF-2: insulin growth factor 2; IGFBP5: insulin growth factor binding protein 5; MSTN: myostatin; MyoD, myogenic differentiation factor 1 ; Myf5: myogenic factor 5 ; AdMyHC: adult isoform of myosin heavy chain; EmbMyHC: embryonic isoform of myosin heavy chain; SlowMyHC: slow myosin heavy chain isoform; NeoMyHC: neonatal isoform of myosin heavy chain; Pax7: paired box 7; NFκB: nuclear factor kappa B; SOD3: superoxide dismutase 3; avian UCP3: avian uncoupling protein 3; Fw: forward primer; Rev: reverse primer.
