# Supplementary material for: Thermal Manipulation during Embryogenesis Has Long-Term Effects on Muscle and Liver Metabolism in Fast-Growing Chickens
Source: PLoS One. 2014 Sep 2;9(9):e105339. doi: 10.1371/journal.pone.0105339 (PMC4152147; doi:10.1371/journal.pone.0105339)
Supplement: Table S2 — Levels of m-RNA expression in the Pectoralis major muscle of 34-day-old broiler chickens. (DOCX) [file pone.0105339.s003.docx]

| **Table S2: Levels of m-RNA expression in the *Pectoralis major* muscle of 34-day-old broiler chickens.** | | | | | | |
| --- | --- | --- | --- | --- | --- | --- |
|  | **C** | **TM** | **CCh** | **TMCh** | ***P*-value**  **Incubation effect** | ***P*-value Challenge(incubation) effect** |
| ADRB2R | 1.43±0.35 | 0.87±0.09 | 1.21±0.08 | 1.17±0.18 | 0.17 | 0.46 |
| ANT | 1.44±0.28 | 0.92±0.09 | 0.77±0.09 | 1.49±0.60 | 0.78 | 0.20 |
| Atrogin-1 | 1.48±0.27AB | 0.87±0.11B | 1.02±0.16AB | 1.95±0.47A | 0.06 | 0.59 |
| DIO2 | 2.14±0.43A | 0.93±0.05B | 0.97±0.16B | 1.37±0.46AB | 0.06 | 0.23 |
| GLUT8 | 1.54±0.14A | 0.92±0.07B | 1.10±0.19AB | 1.26±0.25AB | 0.20 | 0.10 |
| HK2 | 1.14±0.20 | 0.79±0.09 | 0.55±0.07 | 1.53±0.62 | 0.34 | 0.15 |
| IGF-1 | 1.53±0.21A | 0.79±0.08B | 1.04±0.20AB | 1.27±0.32AB | 0.24 | 0.04 |
| LDHA | 2.12±0.51 | 1.40±0.07 | 1.30±0.08 | 1.46±0.14 | 0.30 | 0.11 |
| M-CPT1 | 1.57±0.39 | 1.15±0.04 | 0.77±0.09 | 1.61±0.27 | 0.40 | 0.04 |
| MuRF | 2.20±0.32 | 1.44±0.13 | 1.44±0.21 | 2.09±0.41 | 0.86 | 0.07 |
| AdMyHC | 1.49±0.30AB | 0.88±0.14B | 0.96±0.16AB | 1.65±0.23A | 0.05 | 0.85 |
| EmbMyHC | 1.24±0.28 | 0.99±0.26 | 0.54±0.10 | 1.17±0.29 | 0.44 | 0.13 |
| NeoMyHC | 1.05±0.22 | 1.23±0.17 | 0.98±0.13 | 1.00±0.12 | 0.56 | 0.60 |
| SlowMyHC | 1.23±0.58 | 0.50±0.07 | 0.79±0.11 | 1.04±0.26 | 0.46 | 0.33 |
| Myogenin | 1.28±0.17 | 0.98±0.19 | 0.84±0.22 | 1.67±0.59 | 0.45 | 0.25 |
| NFκB | 1.56±0.25 | 0.81±0.08 | 1.07±0.12 | 1.42±0.40 | 0.42 | 0.10 |
| Pax7 | 1.53±0.19 | 0.82±0.11 | 1.29±0.31 | 1.64±0.55 | 0.60 | 0.22 |
| PPARα | 2.20±0.44 | 1.41±0.07 | 1.35±0.12 | 1.90±0.41 | 0.70 | 0.10 |
| PPARδ | 1.28±0.25 | 0.95±0.05 | 0.98±0.16 | 1.58±0.59 | 0.68 | 0.34 |
| ADRB2R | 1.43±0.35 | 0.87±0.09 | 1.21±0.08 | 1.17±0.18 | 0.17 | 0.46 |
| SOD3 | 0.87±0.26 | 0.50±0.09 | 0.64±0.13 | 1.21±0.65 | 0.54 | 0.35 |

Chickens were incubated or reared in standard conditions (Controls C), thermally-manipulated during embryogenesis and reared in standard conditions (TM), incubated in standard conditions and exposed to heat challenge at 34 d (CCh), or thermally-manipulated during embryogenesis and exposed to heat challenge at 34d (TMCh; n=8 per treatment). Values were standardized using genorm normalization factor calculated from the expression of 18S ribosomal RNA, Cytochrome b and β-actin. ADRB2R: Adrenergic receptor; ANT: adenine nucleotide translocator; Atrogin-1; DIO2: deiodinase 2; GLUT8: glucose transporter 8; HK2: hexokinase 2; IGF-1: insulin growth factor-1; LDHA: lactate dehydrogenase; M-CPT1: muscle carnitine palmitoyltransferase 1; MuRF: muscle specific ubiquitin ligase; AdMyHC: adult isoform of myosin heavy chain; EmbMyHC: embryonic isoform of myosin heavy chain; NeoMyHC: neonatal isoform of myosin heavy chain; SlowMyHC: slow isoform of myosin heavy chain; MSTN: myostatin; NFκB: nuclear factor kappa B; Pax7: paired box 7; PPARα: peroxisome-proliferator-activated-receptor alpha; PPARδ: peroxisome-proliferator-activated-receptor delta; SOD3: Superoxide dismutase 3.
