# Supplementary material for: Thermal Manipulation during Embryogenesis Has Long-Term Effects on Muscle and Liver Metabolism in Fast-Growing Chickens
Source: PLoS One. 2014 Sep 2;9(9):e105339. doi: 10.1371/journal.pone.0105339 (PMC4152147; doi:10.1371/journal.pone.0105339)
Supplement: Table S3 — Levels of m-RNA expression in the livers of 34-day-old broiler chickens. (DOCX) [file pone.0105339.s004.docx]

| **Table S3: Levels of m-RNA expression in the livers of 34-day-old broiler chickens.** | | | | | | |
| --- | --- | --- | --- | --- | --- | --- |
|  | **C** | **TM** | **CCh** | **TMCh** | ***P*-value Incubation effect** | ***P*-value Challenge(incubation) effect** |
| ANT | 1.81±0.32 | 1.73±0.19 | 1.73±0.28 | 1.27±0.12 | 0.27 | 0.41 |
| COX4 | 2.06±0.35 | 1.80±0.22 | 1.40±0.29 | 1.44±0.15 | 0.67 | 0.15 |
| DIO1 | 2.09±0.28 | 1.87±0.18 | 1.43±0.34 | 1.35±0.14 | 0.56 | 0.07 |
| GCK | 0.68±0.09 | 0.69±0.12 | 0.54±0.09 | 0.84±0.31 | 0.39 | 0.72 |
| GLUT8 | 1.61±0.21 | 1.51±0.14 | 1.24±0.25 | 1.08±0.11 | 0.49 | 0.11 |
| HAD | 2.07±0.32 | 1.96±0.19 | 1.69±0.37 | 1.71±0.22 | 0.87 | 0.53 |
| HK1 | 1.40±0.22 | 1.09±0.13 | 1.03±0.20 | 0.88±0.07 | 0.17 | 0.22 |
| HK2 | 1.50±0.20A | 1.35±0.12AB | 0.87±0.18B | 1.25±0.17AB | 0.48 | 0.04 |
| L-CPT1 | 1.43±0.27 | 0.95±0.13 | 1.07±0.22 | 1.13±0.16 | 0.31 | 0.38 |
| LDHA | 1.06±0.11 | 1.21±0.23 | 1.14±0.19 | 1.11±0.14 | 0.74 | 0.87 |
| NFκB | 1.80±0.16 | 2.06±0.31 | 1.30±0.25 | 1.76±0.17 | 0.13 | 0.22 |
| PGC-1α | 0.94±0.34 | 0.71±0.16 | 1.55±0.44 | 1.04±0.17 | 0.24 | 0.28 |
| PPARα | 1.81±0.38 | 1.35±0.13 | 1.71±0.41 | 1.80±0.13 | 0.54 | 0.55 |
| PPARδ | 1.60±0.19 | 1.55±0.23 | 1.10±0.18 | 1.12±0.13 | 0.93 | 0.06 |
| SOD3 | 0.76±0.19 | 0.60±0.17 | 0.47±0.12 | 0.41±0.05 | 0.44 | 0.26 |
| SREBP-1 | 1.49±0.27AB | 1.69±0.60A | 0.79±0.27B | 1.06±0.19AB | 0.34 | 0.03 |

Chickens were incubated and reared in standard conditions (Controls C), thermally-manipulated during embryogenesis and reared in standard conditions (TM), incubated in standard conditions and exposed to heat challenge at 34 d (CCh), or thermally-manipulated during embryogenesis and exposed to heat challenge at 34d (TMCh; n=8 per treatment). Values were standardized using genorm factor calculated from the expression of 18S ribosomal RNA, Cytochrome b and β-actin. ANT: adenine nucleotide translocator; COX4: subunit 4 of cytochrome c oxidase; DIO1: deiodinase 1; GCK: glucokinase; GLUT8: glucose transporter 8; HAD: β-hydroxyacyl-CoA dehydrogenase; HK1: hexokinase 1; L-CPT1: liver isoform of carnitine palmitoyltransferase 1; LDHA: lactate dehydrogenase; NFκB: nuclear factor kappa B; PGC-1α: peroxisome-proliferator-activated receptor coactivator 1 alpha; PPARα: peroxisome-proliferator-activated-receptor alpha; PPARδ: peroxisome-proliferator-activated-receptor delta; SOD3: superoxide dismutase; SREBP-1: Sterol regulatory element-binding protein-1.
