# Supplementary material for: Thermal Manipulation during Embryogenesis Has Long-Term Effects on Muscle and Liver Metabolism in Fast-Growing Chickens
Source: PLoS One. 2014 Sep 2;9(9):e105339. doi: 10.1371/journal.pone.0105339 (PMC4152147; doi:10.1371/journal.pone.0105339)
Supplement: Table S4 — Levels of phosphorylation of kinases in the livers of 34-day-old broiler chickens. (DOCX) [file pone.0105339.s005.docx]

| **Table S4: Levels of phosphorylation of kinases in the livers of 34-day-old broiler chickens.** | | | | | | |
| --- | --- | --- | --- | --- | --- | --- |
|  | **C** | **TM** | **CCh** | **TMCh** | ***P*-value Incubation effect** | ***P*-value Challenge(incubation) effect** |
| p-AMPK/AMPK | 1.04 ± 0.10 | 1.09 ± 0.10 | 1.03 ± 0.10 | 0.91 ± 0.10 | 0.71 | 0.48 |
| p-ERK/ERK | 1.06 ± 0.12 | 1.25 ± 0.12 | 0.86 ± 0.12 | 0.93 ± 0.12 | 0.28 | 0.10 |
| p-p38/p38 | 0.94 ± 0.32 | 1.34 ± 0.32 | 110 ± 0.32 | 1.08 ± 0.32 | 0.56 | 0.79 |

Chickens were incubated and reared in standard conditions (Controls C), thermally-manipulated during embryogenesis and reared in standard conditions (TM), incubated in standard conditions and exposed to heat challenge at 34 d (CCh), or thermally-manipulated during embryogenesis and exposed to heat challenge at 34d (TMCh; n=8 per treatment). All western-blots were performed using anti-vinculin antibody as protein loading control; AMPK: AMP activated protein kinase; ERK: extracellular signal-regulated protein kinase; p38: p38 mitogen-activated protein kinase (p38 MAPK).
